# Surgical Oncologists and Nurses in Breast Cancer Care are Ready to Provide Pre-Test Genetic Counseling

**DOI:** 10.1245/s10434-023-13229-5

**Published:** 2023-02-28

**Authors:** K. Bokkers, E. M. A. Bleiker, C. M. Aalfs, T. van Dalen, M. E. Velthuizen, P. Duijveman, R. H. Sijmons, W. Koole, E. J. P. Schoenmaeckers, M. G. E. M. Ausems

**Affiliations:** 1 Division Laboratories, Pharmacy and Biomedical Genetics, Department of Genetics, University Medical Center Utrecht, Utrecht, The Netherlands; 2 Division of Psychosocial Research and Epidemiology, The Netherlands Cancer Institute, Amsterdam, The Netherlands; 3 Family Cancer Clinic, The Netherlands Cancer Institute, Amsterdam, The Netherlands; 4 Department of Clinical Genetics, Leiden University Medical Center, Leiden, The Netherlands; 5 Department of surgery, Diakonessenhuis Utrecht, Utrecht, The Netherlands; 6 Department of surgery, St. Antonius Hospital, Utrecht, The Netherlands; 7 Department of Genetics, University Medical Center Groningen, University of Groningen, Groningen, The Netherlands; 8 Department of surgery, Meander Medical Center, Amersfoort, The Netherlands

## Abstract

**Background:**

Pre-test genetic counseling for patients with breast cancer is increasingly being provided by nongenetic healthcare professionals. We evaluated the attitudes, knowledge, and self-efficacy of surgeons, oncologists, and nurses regarding mainstream genetic testing and the feasibility to incorporate pre-test genetic counseling into routine care.

**Methods:**

We offered an online training to healthcare professionals from 13 hospitals and implemented a mainstream genetic testing pathway in 11/13 (85%) hospitals. Questionnaires were sent before (*T*0) and 6 months after (*T*1) completing the training. Those who did not complete the training received a questionnaire to assess their motivations.

**Results:**

In 11 hospitals, 80 (65%) healthcare professionals completed the training, of whom 70 (88%) completed both questionnaires. The attitudes, (perceived) knowledge and self-efficacy of healthcare professionals were high both at baseline and 6 months after completing the training. After 6 months, their perceived knowledge about the advantages and disadvantages of a genetic test and implications for family members had significantly improved (*p *= 0.012 and *p *= 0.021, respectively). For the majority (89%), the time investment for pre-test genetic counseling was less than 15 min per patient and as expected or better. Healthcare professionals considered the total time investment feasible to incorporate mainstream genetic testing into their daily practice. The main barrier to complete the training was lack of time. The online training was considered useful, with a rating of 8/10.

**Conclusion:**

Surgical oncologists and nurses in breast cancer care feel well-equipped and motivated to provide pre-test genetic counseling after completion of an online training module.

**Supplementary Information:**

The online version contains supplementary material available at 10.1245/s10434-023-13229-5.

In 5–10% of breast cancer cases, a germline pathogenic variant in one of the breast cancer genes can be found.^1–3^ The identification of a hereditary cause may affect both surgical and chemotherapeutic treatment, and may help decision-making for risk reducing options for both patients and family members.^4–6^

Traditionally, pre-test genetic counseling (GC) is provided by genetic healthcare professionals (HCPs) at genetics departments.^7^ However, not all eligible patients are being referred,^8–11^ and in those tested, results are not always available before surgery.^12,13^ In addition, the burden on genetics departments is rising as waiting lists increase.^7,14^

One strategy to offer genetic testing (GT) to a larger proportion of patients and to decrease time to test results, is to implement mainstream GT. In this approach, pre-test GC is being provided by nongenetic HCPs instead of genetic HCPs.^15–17^ Ideally, these initiatives include training, because many HCPs lack knowledge or confidence to offer pre-test GC.^13^^,^^18–21^ Mainstream GT has been shown to be feasible and acceptable for both patients and HCPs.^22^^,^^23^ However, research has focused primarily on ovarian cancer patients. There is limited information on the experiences of HCPs with mainstream GT in breast cancer patients.^17^^,^^24^ To our knowledge, no studies have evaluated attitudes of HCPs before and after the implementation of mainstream GT. In addition, there is limited information on the feasibility to incorporate such a pathway into routine care. Regarding feasibility, previous studies have focused primarily on time investment.

In this study, we invited HCPs in breast cancer care to complete an online training about GT and we implemented a mainstream GT pathway for patients with breast cancer. Nurse specialists, nurses and doctors work closely together in the care pathway of patients with breast cancer; therefore, we included al these disciplines. We evaluated (1) HCPs’ attitudes toward incorporating mainstream GT into their daily practice, their knowledge of GT, and self-efficacy to offer pre-test GC both before and after implementing mainstream GT; (2) the feasibility for HCPs to incorporate mainstream GT into their daily practice; and (3) HCPs’ experiences with our online training module and their reasons for not completing the training.

## Methods

### Implementation of Mainstream Genetic Testing

#### Needs and Preferences of HCPs

To map the needs and preferences of HCPs involved in breast cancer care, we organized two multidisciplinary focus group meetings. These included surgical oncologists, nurse specialists, a medical oncologist, a radiation oncologist, a clinical geneticist, a genetic counselor, a social worker from the genetics department, a psychologist, and a patient advocate. Based on the discussion points from the first focus group meeting, we performed an online questionnaire among all HCPs in breast cancer care in the service area of the UMC Utrecht genetics department (Supplementary Materials). The results of this survey were discussed during the second focus group meeting.

#### Online Training Module

The online training module consisted of four short films (duration between 7.5 and 15.5 min, Supplementary Materials). Our training module was adapted from the module we developed earlier for HCPs involved in ovarian cancer care.^25^

#### Mainstream Genetic Testing Pathway

**Fig. 1:**
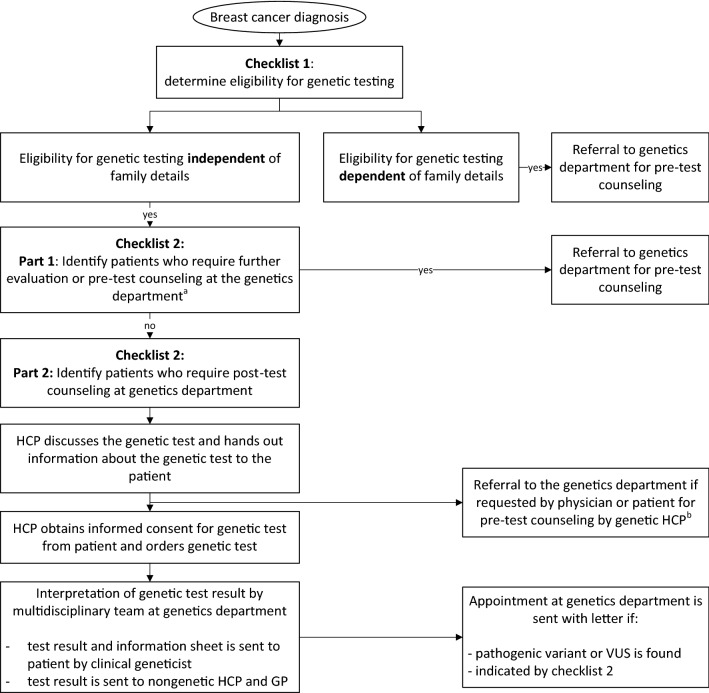
Flow-chart for mainstream genetic testing in patients with breast cancer. *HCP* healthcare professional, *GP* general practitioner, *VUS* variant of uncertain clinical significance ^a^Further genetic evaluation and/or counseling at the genetics department prior to testing, e.g., for additional genetic testing of the *TP53* gene or targeted genetic testing of a known familial pathogenic variant. ^b^Referral to the genetics department for pre-test genetic counseling was optional if requested by the patient or nongenetic healthcare professional (e.g., if the patient had questions that the nongenetic healthcare professional could not answer)

Based on the outcomes of the focus group meetings and survey, we developed a mainstream GT pathway for breast cancer care (Fig. 1). This pathway was adapted from the one developed in the Mainstreaming Cancer Genetics program,^16^ and the pathway we previously developed for ovarian cancer.^25^

Any HCP involved in the treatment of patients with breast cancer could provide pre-test GC themselves when they had completed the training, and patients (1) were eligible for GT based on patient characteristics and independent of family cancer history, and (2) did not require further genetic evaluation and/or counseling at the genetics department prior to testing. These criteria were assessed by the HCP by completing two checklists (Supplementary Materials). If eligible for mainstream GT, HCPs provided pre-test GC and handed out an information sheet to the patient. Patients who consented to GT completed a written consent form and the HCP ordered the genetic test. The gene panel consisted of the genes *BRCA1, BRCA2, CHEK2, PALB2*, and *ATM.* The genetics department sent the test result in a letter to the patient, the HCP who ordered the test, and the patients’ general practitioner. An appointment at the genetics department was only added in case a (likely) pathogenic variant or variant of uncertain clinical significance (VUS) was found or if there was a reason for additional post-test GC based on the second checklist.

#### Implementation of Mainstream Genetic Testing

We implemented our mainstream pathway in nine hospitals in our region: one academic hospital and eight nonacademic teaching hospitals in a stepwise manner (between September 2019 and February 2021). From May 2021 to September 2021, the pathway was also introduced in four nonacademic teaching hospitals in the Northern region of the Netherlands and implemented in two of them during our study period. Due to logistical reasons, the pathway was implemented in the other two hospitals at a later stage, after our study had ended (March 2022). We organized a kick-off meeting in each hospital to introduce our new workflow. Subsequently, HCPs received personal login codes for the training. All HCPs who completed the training received a manual with information and forms to provide pre-test GC and GT. Before the implementation of our pathway, all patients with breast cancer needed to be referred to a genetic HCP for pre-test genetic counseling.

### Study Procedure

A prospective follow-up design was used. Two questionnaires were sent to participating HCPs to assess their attitudes and experiences. The first questionnaire was completed before accessing the online training (*T*0). After 6 months, the second questionnaire (*T*1) was sent to HCPs who completed the training. Items of the questionnaires are shown in the Supplementary Materials.

#### Attitudes, Perceived Knowledge, Self-Efficacy, and Knowledge

Both the T0 and T1 questionnaire contained 13 self-developed statements to evaluate HCPs’ attitudes regarding mainstream genetic testing (four statements), perceived knowledge (three statements), and self-efficacy (i.e., confidence in providing pre-test genetic counseling, five statements).^25^ The second questionnaire contained two extra statements regarding HCPs’ attitudes. All statements were rated using a five-point Likert rating scale, ranging from strongly disagree to strongly agree.

Knowledge was assessed with four self-developed multiple-choice questions, comparable to the questions in our previous study.^25^ In addition, knowledge was assessed with five statements adapted from Claes et al.,^26^ which could be answered with “true,” “false,” or “I do not know.”

#### Feasibility of Mainstream Genetic Testing

Feasibility was assessed based on HCPs’ (1) estimated time investment to discuss and order GT, (2) need for additional appointments for pre-test GC, (3) experiences with the supporting resources to provide pre-test GC, and (4) reasons for not discussing GT with all eligible patients.

#### Evaluation of Online Training Module and Reasons for Not Completing the Module

A short questionnaire was completed after viewing each film and at the end to evaluate the training module. These included questions on duration, usefulness of the content and online format, and level of difficulty, using five-point Likert scales. In addition, HCPs rated each film and the entire training on a scale of 1–10. After 6 months, we asked HCPs whether, in retrospect, they had missed information in the online training.

HCPs who did not complete the online training received a questionnaire to assess their motivations for not starting or completing the training, consisting of three to ten multiple choice questions.

### Statistics

All analyses were performed using IBM SPSS statistics 26.0.0.1. Descriptive statistics were used to describe HCPs’ characteristics, reasons for not discussing the option of GT, time investment, and need for additional appointments to provide pre-test GC. We compared the characteristics between HCPs using the independent *t*-test or Mann–Whitney *U*-test for continuous variables and the chi-square test or Fisher’s exact test for categorical variables. Attitude, perceived knowledge, and self-efficacy were recoded into positive (agree or strongly agree) and negative (neutral, disagree, or strongly disagree). We compared these statements between *T*0 and *T*1 using the Wilcoxon signed-rank test for paired analysis to assess whether their answers had changed (i.e., no change, from negative to positive, or vice versa). The knowledge questions were also compared between *T*0 and *T*1 using the Wilcoxon signed-rank test for paired analysis, both for the individual questions and the total score of all combined questions (possible scores between 0 and 9). We included the answers of the *T*0 questionnaire only if HCPs also completed the *T*1 questionnaire. A *p*-value < 0.05 was considered statistically significant.

### Ethical Approval

This study was reviewed by the Medical Review Ethics Committee (MREC) of the UMC Utrecht in August 2019 and the Medical Research Involving Human Acts (WMO) did not apply to our study and therefore official approval by the MREC was not necessary.

## Results

### Participants

**Fig. 2:**
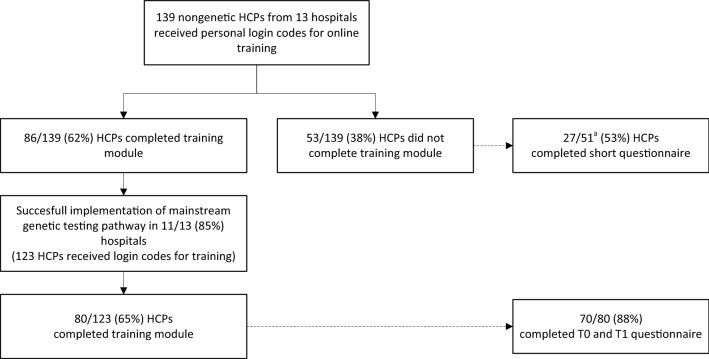
Participation of nongenetic healthcare professionals in online training and questionnaire study. *HCP* healthcare professional ^a^Two healthcare professionals were not sent the short questionnaire

Figure 2 shows the number of HCPs who completed the training and participated in the questionnaire study. In total, 83% of the invited nurses (*n *= 50/60) completed the training versus 46% of invited doctors (*n *= 36/79, *p *= 0.000). In addition, 71% of HCPs working in a surgical department (*n *= 67/94) completed the training versus 42% of HCPs working in an oncology or radiotherapy department (*n *= 19/45, *p *= 0.001).

Table 1 presents the basic characteristics of all HCPs who completed both questionnaires. The majority of participants were female (84%) with an average age of 48 years, working as a nurse in a surgical department (51%), and with varying experience in breast cancer care. Of all HCPs who received the T1 questionnaire (*n *= 80), 94% of nurses (*n *= 43/46) versus 77% of doctors (*n *= 26/34) completed this questionnaire (*p *= 0.047).

**Table 1:** Characteristics of participating nongenetic healthcare professionals

Characteristics	Total group *n *= 70
*Age in years, mean (SD)*	48.0 (9.9)
*Sex, n (%)*	
Female	59 (84.3)
Male	11 (15.7)
*Disciplines,* *n* *(%)*	
* Surgical department*	53 (75.7)
Surgical oncologist	16 (30.2)
Nurse specialist/physician assistant/nurse (in training)	36 (67.9)
Other	1 (1.9)
* Oncology department*	17 (24.3)
Medical oncologist	10 (58.8)
Nurse specialist/physician assistant/nurse (in training)	7 (41.2)
*Years working in breast cancer care, n (%)*	
< 5	14 (20.0)
5–10	17 (24.3)
10–15	15 (21.4)
> 15	24 (34.3)

### Attitudes, Perceived Knowledge, Self-Efficacy, and Knowledge

Table 2 presents HCPs’ attitude toward mainstream GT, perceived knowledge of GT, and self-efficacy to discuss and order GT, both at baseline (*T*0) and after 6 months (*T*1). The majority of HCPs “agreed” or “strongly agreed” with all statements. Only a narrow majority felt confident to explain the differences between germline and tumor testing (53% at *T*0 and 55% at *T*1). There were no significant differences in attitude and self-efficacy before and 6 months after completing the training. Reasons for not having a positive attitude toward mainstream GT are shown in the Supplementary Materials. Perceived knowledge of the advantages and disadvantages of GT and the importance of GT to family members had improved significantly 6 months after completing the training (*p *= 0.012 and *p *= 0.021, respectively).

**Table 2:** Statements evaluating HCPs’ attitude, perceived knowledge, and self-efficacy at baseline (*T*0) and 6 months (*T*1) after completing the training module, *n *= 70

Statements	*T*0	*T*1	Paired comparison between *T*0 and *T*1			
	(strongly) agree	(strongly) agree	+ *n* (%)	− *n* (%)	= *n* (%)	*p*-Value
	*n* (%)	*n* (%)				
*Attitude*						
I am positive toward offering a genetic test myself	48 (68.6)	52 (74.3)	7 (10.0)	3 (4.3)^a^	60 (85.7)	ns
It is important for patients to have a choice whether or not to have a genetic test performed	65 (97.0)^b^	65 (97.0)^b^	1 (1.5)	1 (1.5)	65 (97.0)	ns
It is important to offer genetic testing immediately after diagnosing breast cancer	41 (62.1)^c^	39 (59.1)^c^	8 (12.1)	10 (15.2)	48 (72.7)	ns
It is important that all patients with breast cancer who are eligible for genetic testing have access to genetic testing	65 (98.5)^c^	65 (98.5)^c^	1 (1.5)	1 (1.5)	64 (97.0)	ns
It is important to pay attention to the psychosocial consequences of genetic testing when discussing genetic testing	66 (100)^c^	65 (98.5)^c^	0	1 (1.5)	65 (98.5)	ns
Surgical oncologists, radiation oncologists, medical oncologists**,** and nurse specialists are capable of offering pre-test genetic counseling and request genetic testing themselves after completing an online training module	Not asked	43 (65)^c^	N/A	N/A	N/A	N/A
Surgical oncologists, radiation oncologists, medical oncologists**,** and nurse specialists are capable of offering pre-test genetic counseling and request genetic testing themselves without completing an online training module	Not asked	1 (2)^c^	N/A	N/A	N/A	N/A
*Perceived knowledge*						
I understand the advantages and disadvantages of a genetic test	47 (70.1)^b^	58 (86.6)^b^	15 (22.4)	4 (6.0)	48 (71.6)	0.012*
I understand the importance of genetic testing for patients with breast cancer	63 (95.5)^c^	66 (100)^c^	3 (4.6)	0	63 (95.4)	ns
I understand the importance of genetic testing for family members of patients with breast cancer	55 (83.3)^c^	63 (95.5)^c^	10 (15.2)	2 (3.0)	54 (81.8)	0.021*
*Self-efficacy*						
I am confident that I can discuss the advantages and disadvantages of a genetic test	46 (69.7)^c^	53 (80.3)^c^	13 (19.7)	6 (9.1)	47 (71.2)	ns
I am confident that I am able to discuss a genetic test with all patients with breast cancer directly after diagnosing breast cancer	49 (74.2)^c^	45 (68.2)^c^	6 (9.1)	10 (15.1)	50 (75.8)	ns
I am confident that I am able to order a genetic test myself	54 (81.8)^c^	54 (81.8)^c^	7 (10.6)	7 (10.6)	52 (78.8)	ns
I am confident that I am able to recognize psychosocial problems in patients and subsequently refer patients to a specialist social worker	55 (83.3)^c^	53 (80.3)^c^	5 (7.6)	7 (10.6)	54 (81.8)	ns
I am confident that I am able to explain what genetic testing in tumor tissue entails and what the differences are with genetic testing in blood samples	35 (53.0)^c^	36 (54.5)^c^	7 (10.6)	6 (9.1)	53 (80.3)	ns

*N/A* not applicable, *ns* not significant, *p<0.05

Scores consisted of five options: (1) strongly disagree, (2) disagree, (3) agree nor disagree, (4) agree, (5) strongly agree. Paired analyses with Wilcoxon signed rank test comparing whether patients were positive (agree/strongly agree) or negative (agree, nor disagree/disagree/strongly disagree)

+ negative at T0 and positive at T1,− positive at T0 and negative at T1,= positive or negative in both questionnaires. ^a^The reasons for three HCPs to have a more negative attitude were pre-test genetic counseling was too time consuming, limited experience with mainstream genetic testing, and there was no appropriate time during a consultation to offer pre-test genetic counseling. ^b^Total number of paired measurements was *n *= 67. ^c^Total number of paired measurements was *n *= 66

Table 3 presents the number of HCPs that answered the knowledge questions correctly and their average total score both at baseline and 6 months after completing the training. With paired analyses, there were no significant difference in knowledge for any of the individual knowledge questions and the total scores of all questions combined.

**Table 3:** Number of correct answers of healthcare professionals to knowledge questions before (*T*0) and 6 months after completing the training module (*T*1), *n *= 68

Questions	*T*0	*T*1	Comparison (paired) between *T*0 and *T*1			
	Correct answer *n* (%)	Correct answer *n* (%)	+ *n* (%)	− *n* (%)	= *n* (%)	*p*-Value
What is the prevalence of a pathogenic variant in one of the breast cancer genes?	36 (52.9)	32 (47.1)	12 (17.7)	16 (23.5)	40 (58.8)	ns
What is the meaning of a pathogenic variant in one of the breast cancer genes found only with a tumor test?	45 (66.2)	46 (67.6)	13 (19.1)	12 (17.7)	43 (63.2)	ns
What is the meaning of a pathogenic variant in one of the breast cancer genes found only with a blood test?	55 (80.9)	59 (86.8)	10 (14.7)	6 (8.8)	52 (76.5)	ns
What are the implications of a pathogenic variant in one of the breast cancer genes for obtaining disability or life insurance?	55 (80.9)	60 (88.2)	8 (11.8)	3 (4.4)	57 (83.8)	ns
All women with a pathogenic variant (gene alteration) in a breast cancer gene will someday develop breast cancer	68 (100)	68 (100)	0 (0)	0 (0)	68 (100)	ns
A woman without a pathogenic variant (gene alteration) in a breast cancer gene can still develop breast cancer	68 (100)	66 (97.1)	0 (0)	2 (2.9)	66 (97.1)	ns
A woman with a pathogenic variant (gene alteration) in breast cancer gene can pass this alteration on to her children	67 (98.5)	68 (100)	1 (1.5)	0 (0)	67 (98.5)	ns
A woman may have inherited a pathogenic variant (gene alteration) in a breast cancer gene from her father	68 (100)	68 (100)	0 (0)	0 (0)	68 (100)	ns
A woman who has a sister with a pathogenic variant (gene alteration) in a breast cancer gene has a 50% chance (one in two) of having this gene alteration as well	50 (74.6)^a^	53 (79.1)^a^	11 (16.4)^a^	8 (11.9)^a^	48 (71.7)^a^	ns
Total score, median (min–max)	8.0 (5–9)^a^	8.0 (5–9)^a^	21 (31.3)^a,b^	18 (26.9)^a,b^	28 (41.8)^a,b^	ns

*ns* not significant, + number of healthcare professionals who answered the individual questions incorrect at *T*0 and correct at *T*1 or had a higher total score at *T*1 than at *T*0,− number of healthcare professionals who answered the individual questions correct at *T*0 and incorrect at *T*1 or had a lower total score at *T*1 than at *T*0,= number of HCPs who had the same answer (correct or incorrect) at *T*0 and *T*1 for the individual questions or had the same total score at *T*1 as at *T*0. ^a^*n *= 67, ^b^compared with those HCPs whose scores remained the same or improved, a significant higher proportion of HCPs working in a surgical department (*n *= 17/50, 34%) had a lower overall score after 6 months than oncological HCPs (*n *= 1/17, 6%, *p *= 0.028)

### Feasibility of Mainstream Genetic Testing

In total, 76% of HCPs (*n *= 53/70) had provided pre-test GC and/or ordered a genetic test (on average five HCPs per hospital). These HCPs included 30 nurses (57%) working in a surgical department, 15 surgical oncologists (28%), six nurses working in an oncology department (11%), and two medical oncologists (4%).

The majority of HCPs (72%) both performed pre-test GC and ordered genetic tests. The time investment for pre-test counseling was less than 15 min per patient for 89% of HCPs (*n *= 40/45). This was as or better than expected for 91% of HCPs (*n *= 41/45). The time investment to order a genetic test was less than 15 min for 86% of HCPs (*n* = 37/43). This was as or better than expected for 70% of HCPs (*n *= 30/43). The total time investment for all tasks was feasible for 83% of HCPs (*n *= 44/53). In total, 34% of HCPs (*n *= 18/53) needed to schedule additional appointments to provide pre-test GC, 17% of HCPs (*n *= 9/53) received questions they could not answer, and more than 88% considered the supporting material as useful (Supplementary Materials).

**Fig. 3:**
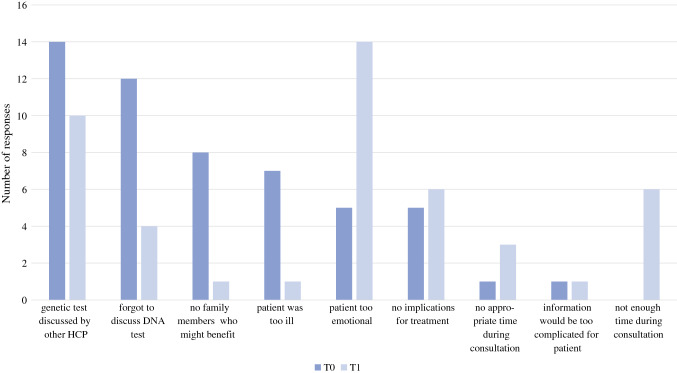
Reasons of nongenetic healthcare professionals for not discussing genetic testing before (T0) and 6 months after completing the online training module (T1), *n *= 69. Multiple reasons could be given

The main reason for not discussing GT with patients before the online training (T0) was that HCPs forgot to discuss it, whereas after 6 months (T1) the main reason was that patients were too emotional (Fig. 3).

In total, 25% of HCPs (*n *= 17/67) had not provided pre-test GC and/or requested GT during the 6 months after completing the training. They consisted of eight medical oncologists (47%), six nurses (35%) working in a surgical department, two surgical oncologists (12%), and one nurse (6%) working in an oncology department. The main reasons were that HCPs did not encounter patients eligible for GT (*n *= 11), colleagues discussed and/or ordered GT (*n *= 5), there was not enough time (*n *= 1), or no appropriate moment (*n *= 1) during consultations.

### Evaluation of Online Training Module and Reasons for Not Completing the Module

The median rating for the training was eight out of ten. The majority of HCPs considered the training to be useful (95%), appreciated the online format (99%), and considered the level of difficulty and duration to be exactly right (80% and 78%, respectively) (Supplementary materials). Six months after completing the training, 11% of HCPs (*n *= 6/53) indicated that, in retrospect, they had missed information in the training.

We received 27/51 (53%) questionnaires from HCPs who did not complete the online training. Their main reasons were having no time or being too busy (*n *= 17/27, 63%).

## Discussion

In this study, we implemented mainstream GT for patients with breast cancer and show that surgical oncologists, nurse specialists, and nurses in breast cancer care are ready to provide pre-test GC.

### Attitudes, Perceived Knowledge, Self-Efficacy and Knowledge

HCPs had a positive attitude, high self-efficacy, and high perceived knowledge both before and after implementing our mainstream pathway. Previous research has shown conflicting results, with both HCPs having a positive attitude toward mainstream GT,^17^^,^^18^^,^^24^ but also expressing concern about their ability to provide adequate pre-test GC.^13^^,^^19^^,^^21^ A possible explanation for the positive attitude and high participation rate in our training is the close involvement of HCPs in shaping our new pathway. This allowed them to raise concerns and consider the new pathway.

After 6 months, the self-perceived knowledge of HCPs had improved regarding the advantages and disadvantages of GT and the consequences for family members. Therefore, we believe that our training had a positive influence on HCPs’ confidence to provide pre-test GC as described in previous research.^27^^,^^28^ Other studies evaluating the experiences of HCPs with mainstream GT have also shown that these HCPs are confident to consent patients for GT.^17^^,^^24^ In addition, we found an improvement in self-perceived knowledge regarding the advantages and disadvantages of GT among gynecologic HCPs participating in mainstream GT for ovarian cancer, although due to the relatively small sample size, this was not a significant difference.^25^ Our training probably contributed to this effect, although we did not evaluate these outcomes in HCPs who did not complete a training module.

We did not measure an objective increase in knowledge after completing the training. However, overall knowledge scores were already high at baseline, suggesting a ceiling effect. Interestingly, one in four HCPs had worse overall knowledge scores 6 months after completing the training than at baseline. However, this decrease in knowledge was not significant. Especially, questions about the prevalence of detecting a pathogenic variant and the difference between blood and tumor GT seemed to have contributed to this. The question about the prevalence of a pathogenic variant might have been too specific, whereas the question about tumor GT might still be too difficult for HCPs in breast cancer care. Tumor testing is currently not used as a pre-screen for germline genetic testing, as it is in ovarian cancer care.^29^ This is consistent with HCPs’ self-efficacy; only a small majority felt confident to discuss the difference between blood and tumor testing. This suggests a specific training need when tumor testing becomes more prominent in the future. To our knowledge, no other studies have evaluated knowledge after implementing mainstream GT.

### Feasibility of Mainstream Genetic Testing

Timing of pre-test GC can be challenging, because at time of diagnosis emotions are high and patients already receive a lot of information.^30^^,^^31^ This is also shown in the relatively high proportion of HCPs (33%) who did not feel confident to discuss GT directly after diagnosis. However, surgical decisions may require a timely test result.^12^ In our study, the majority of HCPs managed to discuss GT within 15 min, which they considered as or better than expected. This timeframe is comparable with previous research in which HCPs needed between 8 and 20 min for pre-test GC.^22^ The time to order a genetic test in our study was also less than 15 min for most HCPs. Although they still considered this time investment as or better than expected, the time to order a genetic test was one of the main reasons for a negative attitude. Therefore, this time investment should be reduced, for example, by delegating these tasks to outpatient staff. Most importantly, however, the total time investment required by HCPs to discuss and/or order GT was feasible for more than 80% of HCPs. This is in line with previous research in which HCPs agreed that it was possible to discuss GT within the timeframe of a consultation.^22^

Notably, especially nurse specialists and nurses were closely involved in our mainstream genetic testing pathway. Before the implementation of this pathway, these HCPs were already actively involved in the referral of eligible patients to the genetics department under the supervision of surgical oncologists. This study shows that nurse specialists and nurses are well-equipped to perform these tasks and may play an important role in the implementation of a mainstream genetic testing pathway.

### Evaluation of Online Training Module and Reasons for Not Completing the Module

The majority of HCPs appreciated our training module, which is consistent with previous research.^22^ However, we did see a wide variation in overall appreciation, and some HCPs considered our training too easy. We included a wide variety of HCPs (nurses, nurse specialists, doctors) from different departments, which might explain differences in training needs.^32^^,^^33^ Therefore, it might be useful to develop a more tailored training for each discipline. Only one HCP agreed that HCPs were capable of providing mainstream GT without training. This indicates that the HCPs in this study considered training a prerequisite for providing mainstream GT.

The main barrier for HCPs to refrain from participating was lack of time. These HCPs consisted mainly of doctors (e.g., surgical oncologists and medical oncologists). This implies that the success of mainstream GT mainly depends on the involvement of dedicated nurses and nurse specialists.

### Strengths and Limitations

A strength of this multicenter study is the large sample size, high participation rate, and the before-and-after design. Also, prior to the development of our training module and mainstream GT pathway, we performed a needs assessment among all HCPs involved. We believe that the high participation of HCPs can partly be explained by the close collaboration both during the development and implementation phases.

A limitation is that we did not use validated questionnaires since these were non-existent. In addition, the majority of participating HCPs worked in a surgical department. Therefore, our conclusions cannot be generalized for all HCPs in breast cancer care. Although we invited medical oncologists to participate in our mainstream GT pathway, in our study, these HCPs rarely discussed and ordered GT themselves. This is notable, because previous research into mainstream GT in breast cancer care has shown significant involvement of oncologists between 30 and 100%, probably due to differences in care pathways.^19^^,^^34–37^ In our study, patients eligible for GT were initially seen by the surgical team. However, the importance of GT by medical oncologists is expected to increase due to the rise of PARP-inhibiting therapies in carriers of germline *BRCA1/2* pathogenic variants.^38^ Future research should focus on experiences of medical oncologists and also on the impact of mainstream genetic testing on genetic testing rates.

## Conclusion

This study shows that HCPs working in a surgical department (i.e., surgical oncologists, nurse specialists, and nurses) have a positive attitude, feel confident, and capable to provide pre-test GC to patients with breast cancer. In addition, it is feasible for them to incorporate these tasks into their routine work.

## Supplementary Information

Below is the link to the electronic supplementary material.

Supplementary file1 (PDF 1099 KB)
