# Supplementary material for: Surgical Oncologists and Nurses in Breast Cancer Care are Ready to Provide Pre-Test Genetic Counseling
Source: Ann Surg Oncol. 2023 Feb 28;30(6):3248–58. doi: 10.1245/s10434-023-13229-5 (PMC10175452; doi:10.1245/s10434-023-13229-5)
Supplement: Supplementary file 1 — Supplementary file1 (PDF 1099 KB) [file 10434_2023_13229_MOESM1_ESM.pdf]

Supplementary Material

Inhoud

Survey regarding needs assessment of nongenetic healthcare professionals involved in breast cancer care 2

Supplementary Table 1. Characteristics of participating nongenetic healthcare professionals from nine hospitals 2

Supplementary Table 2. Results of Survey, n = 51........ 3

Supporting information regarding online training module........ 5

Content of online training module 5

Supplementary Table 3. Evaluation of overall online training module, n = 85 7

Supplementary Table 4. Reasons for not completing the training module, n = 27/51 (52.9%) 7

Checklists to identify eligible patients for genetic testing and post-test counseling........ 8

Checklist 1: Eligibility criteria for genetic testing in breast cancer........ 9

Checklist 2: referral to genetics department 10

Content of questionnaires........ 11

Supplementary Table 5. Overview of questions in the T0 and T1 questionnaire 11

Reasons for not having a positive attitude toward mainstream genetic testing........ 12

Supplementary Figure 1. Reasons of nongenetic healthcare professionals in breast cancer care for not having a positive attitude toward mainstream genetic testing before (T0) and 6 months after completing the training module (T1) 12

Additional information regarding feasibility for nongenetic HCPs to incorporate germline genetic testing into routine care of breast cancer patients........ 13

Supplementary Table 6. Additional appointments for pre-test genetic counseling, receiving unanswerable questions from patients, usefulness of supporting resources 13

Supplementary Table 7. Performance of tasks and time investment for providing pre-test genetic counseling and ordering a genetic test 14

Survey regarding needs assessment of nongenetic healthcare professionals involved in breast cancer care

Response: 51/89 (57%)

Supplementary Table 1. Characteristics of participating nongenetic healthcare professionals from nine hospitals

**Characteristics**	**Total group, n = 51**<br>n (%)
Disciplines,	
- Nurse specialist/physician assistant (in training)	17 (33.3)
- Surgical oncologist	14 (27.5)
- Medical oncologist	11 (21.6)
- Nurse	7 (13.7)
- Radiation oncologist	2 (3.9)
Hospital, n (%)	
- Non-academic teaching hospital	42 (82.4)
- Academic hospital	9 (17.6)
Years working in breast cancer care, n (%)	
- < 5	13 (25.5)
- 5 – 10	19 (37.2)
- 10 – 15	8 (15.7)
- > 15	11 (21.6)

Supplementary Table 2. Results of Survey, n = 51

Questions	Response categories	Responses n (%)
For which genetic testing eligibility criteria do you want to offer pre-test counseling and order genetic testing yourself?	- Offer mainstream genetic testing to all patients eligible for genetic testing	13 (26)
	- Offer mainstream genetic testing only to a selected group: patients with breast cancer < 40 years and triple negative breast cancer < 60 years	16 (31)
	- Initially start with mainstream genetic testing in a selected group (bullet 2) and at a later stage expand to all eligibility criteria for genetic testing	21 (41)
	- I do not know	1 (2)
	- No preference	0
If mainstreaming is not applied (immediately) to all eligibility criteria, what is your preference for patients who are eligible for genetic testing but not for mainstream genetic testing?	- Referral of these patients to the genetics department	36 (71)
	- The medical specialist/nurse specialist provides general information about genetic testing, arranges for blood storage and conducts a telephone consultation with a genetic healthcare professional	13 (25)
	- I do not know	1 (2)
	- No preference	1 (2)
Do you think the medical specialist/nurse specialist should also ask about other cancers/other symptoms related to rarer hereditary causes of breast cancer?	- Yes	28 (55)
	- No	10 (20)
	- I do not know	10 (20)
	- No preference	3 (6)
Are you willing to ask for more comprehensive family information and not just ask about breast, ovarian and prostate cancer in the family?	- Yes	35 (68)
	- No	8 (16)
	- I do not know	5 (10)
	- No preference	3 (6)
Are you willing to measure a skull circumference?	- Yes	14 (27)
	- No	31 (61)
	- I do not know	6 (12)
	- No preference	0
When a pathogenic variant or variant of uncertain clinical significance is found, patients receive post-test counseling at the genetics department to discuss the result and its implications. Which patients do you think	- All patients, regardless of family history	7 (14)
	- Only patients with a family history of breast cancer	2 (4)
	- Only patients with a family history of breast cancer and/or indicators of a rare tumor syndrome	41 (80)

should also receive post-test counseling from a genetics counselor when no pathogenic variant or variant of unknown clinical significance is found in a breast cancer gene?	- No preference<br>- I do not know	0<br>1 (2)
In your opinion, how should duties be divided between medical specialists and nurse specialists with respect to mainstream genetic testing?	- The medical specialist provides pre-test counseling and assesses the family history	2 (4)
	- The nurse (specialist) provides pre-test counseling and assesses the family history	21 (41)
	- The medical specialists provides pre-test counseling and the nurse (specialists) assesses the family history	5 (10)
	- The nurse (specialist) provides pre-test counseling and the medical specialist assesses the family history	1 (2)
	- Both medical specialist and nurse (specialist) perform pre-test counseling and assess the family history	9 (17)
	- Referral of patient to genetics department	3 (6)
	- No preference	8 (16)
	- I do not know	2 (4)

Supporting information regarding online training module

Content of online training module

The training module was evaluated and accredited by the national accreditation bureaus of the scientific organizations for surgical oncologists, medical oncologists, nurses, and nurse specialists as part of the national CME accreditation.

Film 1: Mainstream genetic testing in cancer patients (duration: 7.5 minutes).

This film contains the following elements:

- Short introductory film with a clinical geneticist explaining the term mainstream genetic testing and why this new workflow is important for cancer patients.
- Slides with a voice-over explaining:
    - Short overview of the content of all four films.
    - How cancer develops and how hereditary and environmental factors play a role.
    - The difference between germline and somatic pathogenic variants.
    - The difference between germline genetic testing in blood and genetic testing in tumor tissue.
    - The importance of genetic testing in cancer patients.
    - The difference between diagnostic and predictive genetic testing.
    - A summary of the above.

Film 2: genetic testing in breast cancer patients (duration: 12.5 minutes).

This film contains the following elements:

- Short introductory film with a clinical geneticist giving an overview of the film's content.
- Slides with a voice-over explaining:
    - Prevalence of hereditary causes in breast cancer patients.
    - The eligibility criteria for genetic testing in breast cancer patients, including the relevance of genetic testing in patients with Ashkenazi Jewish ancestry.
    - The prevalence of pathogenic variants in the genes *BRCA1, BRCA2, CHEK2, ATM,* and *PALB2.*
    - Cancer risks associated with pathogenic variants in the genes *BRCA1, BRCA2, CHEK2, PALB2,* and *ATM*.
    - Explanation of life time risks.

- Possible implications of genetic testing for family members (including screening recommendations and the possibility of risk reducing surgery).
- Possible implications on treatment for women carrying a pathogenic variant in one of the breast cancer genes.
- Male breast cancer; chance of carrying a pathogenic variant in one of the breast cancer genes, cancer risks and possible implications of finding a pathogenic variant.
- Pattern of inheritance for pathogenic variants in the genes *BRCA1, BRCA2, CHEK2, PALB2,* and *ATM*.
- Involvement of higher risk of breast cancer in rare syndromes.
- A summary of the above.

<u>Film 3: The new workflow for genetic testing (duration: 8.5 minutes)</u>

This film contains the following elements:

- Short introductory film with a clinical geneticist giving an overview of the film's content.
- Sides with a voice-over explaining:
    - The entire workflow (step-by-step).
    - How to complete the checklist to determine eligibility for genetic testing.
    - How to complete the checklist to determine if the patient is eligible for referral to the genetics department.
    - How to complete the informed consent form.

<u>Film 4: Practical advice on how to discuss genetic testing (duration: 15.5 minutes)</u>

This film contains the following elements:

- Short introductory film with a clinical geneticist giving an overview of the film's content.
- Slides with a voice-over explaining:
    - Communication about and timing of genetic testing.
    - Elements to discuss with a patient, including the duration of a genetic test.
    - Possible outcomes of a genetic test and the implications of these outcomes.
    - Possible implications for insurance.
- Film with:
    - A surgical oncologist and a patient. This shows a simulation consultation on how to offer pre-test counseling for genetic testing.

- A patient who has a pathogenic variant in the *BRCA2* gene. She explains the impact that genetic testing has had on her and her family and why genetic testing is important for any woman with breast cancer.
- A specialist social worker. She explains the impact a pathogenic variant in a *BRCA* gene can have on a patient and her family members, addresses the emotional impact and explains the supportive role of a social worker in the process of genetic testing.

Supplementary Table 3. Evaluation of overall online training module, n = 85

Rating out of 10, median (range)	8 (5 – 10)
Usefulness of online training module, n (%)	
(Reasonably/very) useful	81 (95.3)
Not useful (at all)	4 (4.7)
Level of difficulty, n (%)	
(Much) too high	0 (0)
Exactly right	68 (80.0)
(Much) too low	17 (20.0)
Appreciation of online format, n (%)	
(Fairly/very) pleasant	84 (98.8)
Not pleasant (at all)	1 (1.2)
Duration of online training module, n (%)	
(Much) too long	14 (16.5)
Exactly right	66 (77.6)
(Much) too short	5 (5.9)

Supplementary Table 4. Reasons for not completing the training module, n = 27/51 (52.9%)

	N (%)
Respondents	
- Surgical oncologists	12 (44.4)
- Medical oncologists	9 (33.3)
- Nurses/nurse specialists working in an oncology department	4 (14.8)
- Nurses/nurse specialists working in a surgical department	1 (3.7)
- Radiation oncologists	1 (3.7)
Reasons for not completing the training module (multiple answers could be provided)	
- No time/too busy	17 (63.0)
- Forgotten	8 (29.6)
- Mainstream genetic testing should not be part of my work[a]	7 (25.9)
- Technical problems with training module	3 (11.1)
- Genetic testing is requested by colleagues	2 (7.4)
- Encounter not enough patients eligible for genetic testing to build expertise	1 (3.7)

There were no significant differences between the respondents and non-respondents regarding disciplines or department they worked in.

[a] HCPs who did not consider mainstream genetic testing as part of their work, mainly considered this as part of the work of a surgical nurse/nurse specialist (n = 6) or genetic HCP (n = 4).

Checklists to identify eligible patients for genetic testing and post-test counseling

1

Checklist 1: Eligibility criteria for genetic testing in breast cancer

UMC Utrecht

Patient details	Date of diagnosis: ....<br>Hospital: ....<br>Surgical oncologist:............<br>Nurse (specialist):............<br>Email address: ....<br>Telephone number:............

CHECKLIST risk factors for hereditary cause breast cancer /DCIS	Yes	No
**Male**	☐	☐
**< 40 years of age**	☐	☐
**< 50 years of age and:**		
* bilateral breast cancer (whether or not diagnosed at the same time)	☐	☐
* a first-degree family member with breast cancer < 50 years of age	☐	☐
* a family member with prostate cancer < 60 years of age	☐	☐
* two or more first or second degree family members with breast cancer in the same branch of the family?	☐	☐
**< 60 years of age and:**		
* triple negative breast cancer	☐	☐
**Does patient have:**		
* a family member with a known *BRCA1/2* pathogenic variant	☐	☐
* a history of contralateral breast cancer < 50 years of age	☐	☐
* multiple tumors in one breast with first tumor < 50 years of age	☐	☐
* a first-degree male family member with breast cancer	☐	☐
* two or more first and/or second degree family members with breast cancer of whom at least one < 50 years of age, in the same branch of the family	☐	☐
* a history of ovarian or fallopian tube carcinoma	☐	☐
* a family member with ovarian or fallopian tube carcinoma	☐	☐
* a Jewish ancestry	☐	☐

'yes' for <u>at least 1</u> grey shaded criterion?	➔ continue with **checklist 2** to determine if you can offer pre-test genetic counseling and order genetic testing
'yes' for only white shaded criteria?	➔ refer patient to genetics department and skip **checklist 2.** Pre-test genetic counseling is offered by a genetic healthcare professional
'no' for all eligibility criteria?	➔ patient is not eligible for genetic testing

Have you ordered genetic testing? ☐ Yes ☐ No

In case of a referral, is it urgent? ☐ Yes ☐ No

Please fax this list including all relevant correspondence and pathology report to the genetics department of the University Medical Center Utrecht.

Was a treatment plan discussed with patient? ☐ No ☐ Yes

If yes, which plan? ☐ mastectomy ☐ breast conserving surgery ☐ Neoadjuvant chemotherapy

Is the operation date known? ☐ No ☐ Yes, on ....

Does patient want to postpone the surgery date if the result of the genetic test is not yet known ☐ No ☐ Yes ☐ Not discussed

At which phone number(s) can the patient be reached? .... / ....

Additional information ....

....

....

2

Only complete this checklist if you answered 'yes' for <u>at least one of the grey shaded criteria</u> on checklist 1!

UMC Utrecht

Checklist 2: referral to genetics department

Patient details	Date: ……………………………………………………………………………………… Hospital: ……………………………………………………………………………………… Surgical oncologist:……………………………………………………………………… Nurse (specialist):……………………………………………………………………… Email address: ……………………………………………………………………………… Telephone number:………………………………………………………………………

Does your patient have:	Yes	No
1. breast cancer/DCIS ≤25 years of age?	☐	☐
2. breast cancer ≤35 years of age and Her2neu positive?	☐	☐
3. bilateral *lobular* breast cancer (whether or not diagnosed at the same time) with first diagnosis <50 years?	☐	☐
4. a family member with a known pathogenic variant in one of the breast cancer genes?	☐	☐
5. (a history of) sarcoma, brain tumor or adrenal cortex		
a) in your patient, with first diagnosis <46 years of age for one of these tumors or the breast cancer?	☐	☐
b) in ≥1 first or second degree family member, with diagnosis <46 year for family member or for your patient with breast cancer?	☐	☐

If 'yes for one or more of these questions → refer patient to genetics department for pre-test genetic counseling. You do not have to complete the rest of this checklist.

Does your patient have:	Yes	No
6. a first or second degree family member with breast cancer and is your patient male?	☐	☐
7. a second primary breast cancer/DCIS with first diagnosis <50 years of age?*	☐	☐
8. a first or second degree family member with breast cancer/DCIS and is the average age of diagnosis for patient and family member <50 years of age?	☐	☐
9. ≥2 first and/or second degree family members with breast cancer/DCIS?	☐	☐
10. ≥2 first and/or second degree family members with ovarian cancer? (of whom at least one first degree family member)	☐	☐

You can now offer pre-test genetic counseling and request the DNA test (note: this applies only if the first five questions on this checklist can be answered with 'no' and at least one grey shaded criterion on checklist 1 is 'yes').
If you have checked one or more boxes with "yes," the patient will receive an additional appointment at the genetics department after the results of the DNA test are known. You do not have to do anything for this yourself. This checklist is faxed to the genetics department and can be used as a formal referral document.

** This includes a second primary tumor diagnosed at the same or different times, multiple tumors in the same breast or in both breasts and multicentric breast cancer.*

Please add this checklist to the electronic patient file

Please contact the genetics department if you have any questions about this checklist or if you think there might be a reason to refer your patient and this is not indicated by this checklist. (original checklist included telephone numbers)

Content of questionnaires

Supplementary Table 5. Overview of questions in the T0 and T1 questionnaire

Topics	T0	T1
Background information	X	
Statements to assess		
- Attitude towards mainstream genetic testing	X	X
- Perceived knowledge of genetic testing	X	X
- Self-efficacy to offer mainstream genetic testing	X	X
Questions assessing knowledge of genetic testing	X	X
Feasibility of mainstream genetic testing		
- Performance of tasks, time investment and necessity of additional appointments		X
- Experiences with supporting resources (e.g., usefulness of training or manual, unanswerable questions from patients, missed information)		X
- Reasons for not discussing genetic testing	X	X

T0: Questionnaire before starting the online training module. T1: Questionnaire 6 months after completing the online training module.

Reasons for not having a positive attitude toward mainstream genetic testing

Supplementary Figure 1. Reasons of nongenetic healthcare professionals in breast cancer care for not having a positive attitude toward mainstream genetic testing before (T0) and 6 months after completing the training module (T1)

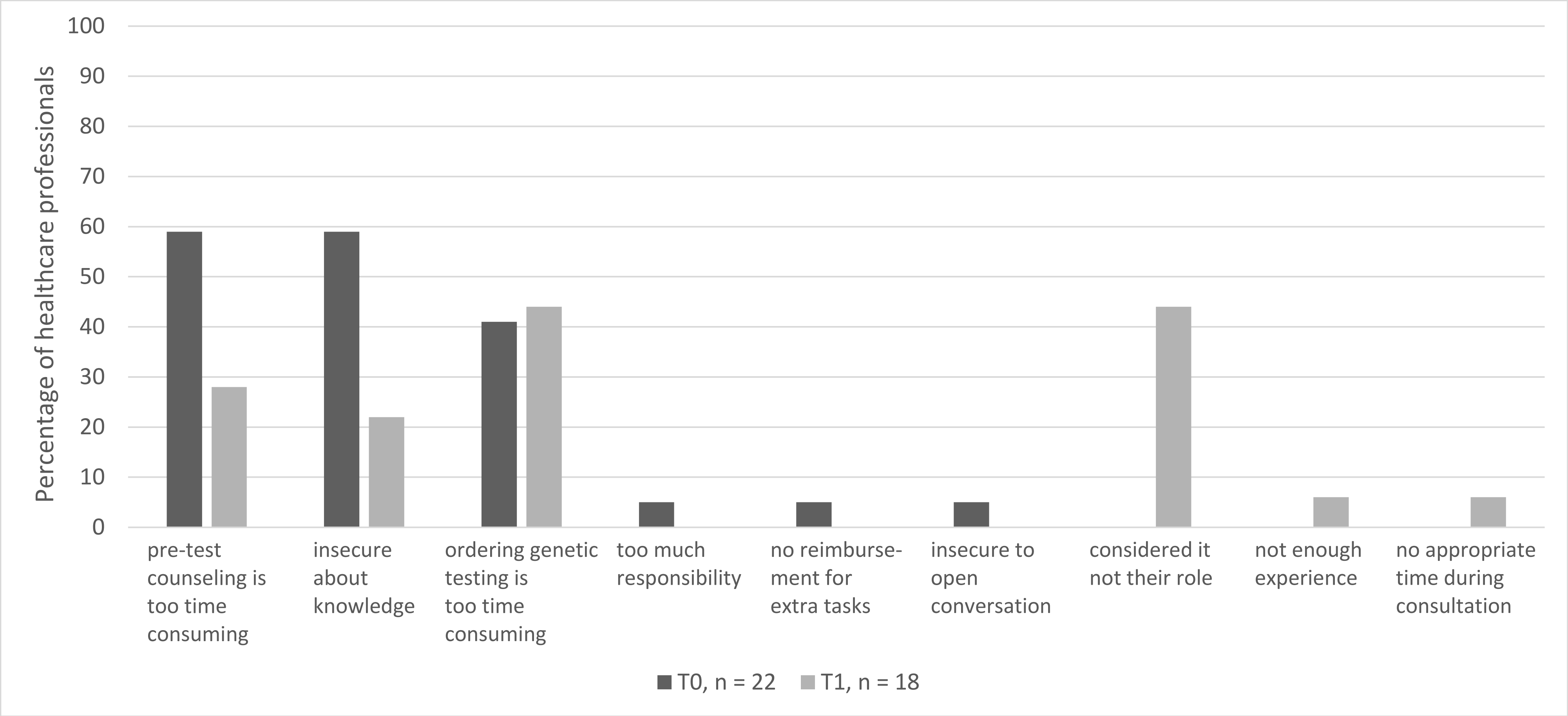

Multiple reasons could be given. Since the T0 questionnaire was completed before experiencing mainstream genetic testing, the answers about time investment for pre-test genetic counseling and ordering genetic testing were based on healthcare professionals' expectations, starting with 'I think…'

Additional information regarding feasibility for nongenetic healthcare professionals to incorporate germline genetic testing into routine care of breast cancer patients

Supplementary Table 6. Additional appointments for pre-test genetic counseling, receiving unanswerable questions from patients, usefulness of supporting resources

	T1, n = 53 n (%)	Reasons/explanations:
**Additional appointments for pre-test genetic counseling**		- Patient needed time to consider the genetic test (n = 15)
- Yes	18 (34.0)	- There was not enough time during one consultation (n = 7)
- No	35 (66.0)	- Patient needed to get clarity regarding family history (n = 1)
**Unanswerable questions**		- Detailed consequences of a pathogenic variant for patient or family members
- Yes	9 (17.0) [a]	(n = 3)
- No	44 (83.0)	- The chance of having an hereditary cause (n = 1)
		- Eligibility criteria (n = 1)
		- How to proceed if there is a more complex family history (n = 2)
		- How to proceed if there is a family history of cancer but no hereditary cause
		- (n = 1)
**I considered it useful to receive information about genetic testing (e.g., the online training module and manual) before providing pre-test genetic counseling myself**		
- (Strongly) agree	49 (92.4)	N/A
- Neutral/ (strongly) disagree	2 (3.8)	
- Missing	2 (3.8)	
**I considered it useful to give written information about genetic testing to the patient after pre-test counseling**		
- (Strongly) agree	47 (88.7)	N/A
- Neutral/ (strongly) disagree	4 (7.5)	
- Missing	2 (3.8)	

N/A: not applicable. [a] *One healthcare professional could not specify which questions were unanswerable.*

Supplementary Table 7. Performance of tasks and time investment for providing pre-test genetic counseling and ordering a genetic test

		n (%)
**Performance of tasks, n = 53**		
-	Pre-test genetic counseling and ordering genetic test	38 (71.7)
-	Pre-test genetic counseling only	7 (13.2)
-	Ordering genetic test only	5 (9.4)
-	Other	3 (5.7)
**Time investment to offer pre-test genetic counseling, n = 45**		
-	< 5 minutes	2 (4.5)
-	5 – 10 minutes	23 (51.1)
-	10 – 15 minutes	15 (33.3)
-	15 – 20 minutes	5 (11.1)
-	> 20 minutes	0 (0)
**Time investment to order genetic test, n = 43**		
-	< 5 minutes	2 (4.7)
-	5 – 10 minutes	18 (41.9)
-	10 – 15 minutes	17 (39.5)
-	15 – 20 minutes	5 (11.6)
-	> 20 minutes	1 (2.3)
